# A RUNX2-Mediated Epigenetic Regulation of the Survival of p53 Defective Cancer Cells

**DOI:** 10.1371/journal.pgen.1005884

**Published:** 2016-02-29

**Authors:** Min Hwa Shin, Yunlong He, Eryney Marrogi, Sajida Piperdi, Ling Ren, Chand Khanna, Richard Gorlick, Chengyu Liu, Jing Huang

**Affiliations:** 1 Cancer and Stem Cell Epigenetics Section, Laboratory of Cancer Biology and Genetics, Center for Cancer Research, National Cancer Institute, Bethesda, Maryland, United States of America; 2 Division of Pediatric Hematology-Oncology, The Children’s Hospital at Montefiore, Bronx, New York, United States of America; 3 Pediatric Oncology Branch, Center for Cancer Research, National Cancer Institute, Bethesda, Maryland, United States of America; 4 Transgenic Core Facility, Division of Intramural Research, National Heart, Lung, and Blood Institute, Bethesda, Maryland, United States of America; St Jude Children's Research Hospital, UNITED STATES

## Abstract

The inactivation of p53 creates a major challenge for inducing apoptosis in cancer cells. An attractive strategy is to identify and subsequently target the survival signals in p53 defective cancer cells. Here we uncover a RUNX2-mediated survival signal in p53 defective cancer cells. The inhibition of this signal induces apoptosis in cancer cells but not non-transformed cells. Using the CRISPR technology, we demonstrate that p53 loss enhances the apoptosis caused by RUNX2 knockdown. Mechanistically, RUNX2 provides the survival signal partially through inducing MYC transcription. Cancer cells have high levels of activating histone marks on the MYC locus and concomitant high MYC expression. RUNX2 knockdown decreases the levels of these histone modifications and the recruitment of the Menin/MLL1 (mixed lineage leukemia 1) complex to the MYC locus. Two inhibitors of the Menin/MLL1 complex induce apoptosis in p53 defective cancer cells. Together, we identify a RUNX2-mediated epigenetic mechanism of the survival of p53 defective cancer cells and provide a proof-of-principle that the inhibition of this epigenetic axis is a promising strategy to kill p53 defective cancer cells.

## Author Summary

Because activated p53 is a potent inducer of apoptosis, several approaches centering on p53 activation are designed for killing cancer cells. However, more than half of human tumors have p53 inactivation, which renders these p53-activating approaches less effective in killing cancer cells. Targeting the survival signals specific to p53 defective cancer cells offers an opportunity to circumvent the challenge of p53 inactivation. In this study, we showed that one such survival signal is the RUNX2 signaling pathway. To investigate the mechanism underlying this survival signal, we used biochemical, genetic, and genomic approaches. The MYC gene was identified as a novel mediator of the pro-survival function of RUNX2. We further studied the regulatory mechanism of MYC by RUNX2 and found that RUNX2 recruits the Menin/MLL1 epigenetic complex to induce the expression of MYC. Using small molecule inhibitors of the Menin/MLL1 complex, we showed that targeting RUNX2/Menin/MLL1/MYC axis is a feasible strategy for killing p53 defective cancer cells. Our study paves the road for the future development of targeted therapies for OS.

## Introduction

Because activated p53 is a potent inducer of apoptosis [1], the activation of p53-dependent apoptosis provides an important molecular basis for killing cancer cells. Chemotherapy and radiotherapy, which cause DNA damage, can activate p53 and induce apoptosis in cancer cells. Many cancer cells have amplification of the MDM2 gene, which encodes an E3 ligase of p53 [2]. Thus, compounds that relieve p53 from the inhibition of MDM2, such as Nutlin and RITA, were sought and discovered [3,4]. Compounds that restore specific p53 mutants to the wild type p53 conformation have also been reported [5]. These p53-centric approaches require either the existence of wild type p53 or a specific p53 mutation. However, when p53 is deleted or mutated in other sites, the pro-apoptotic effects of these approaches diminish. Therefore, the loss-of-function of p53 still represents a big challenge for killing p53 defective cancer cells.

An attractive alternative approach to killing p53 defective cancer cells is to identify survival signals in cancer cells and subsequently inhibit these survival signals [6]. Preferably, the inhibition of this (these) survival signal(s) should induce p53-independent apoptosis. Despite many years of genetic studies and recent genome-wide sequencing endeavors, knowledge of these survival signals in p53 defective cells is largely lacking. It is possible that different cancer types have different survival signals in the absence of p53. One of the cancer types that have high frequency (~90%) of inactivating p53 is osteosarcoma (OS), the most common primary malignant bone tumor in children, adolescents and young adults [7–9]. Thus far, there is no FDA-approved targeted therapy for OS cells. The current standards of care are neoadjuvant chemotherapy followed surgery and adjuvant chemotherapy [10]. The tumor suppressive function of p53 in OS is conserved between human and mouse. Li-Fraumeni syndrome patients who carry p53 mutations have a high risk of developing various cancers including osteosarcoma [11]. Mice with p53 heterozygous deletion develop a high incidence of OS [12]. Thus, OS cells are a good model to study the survival signals of p53 defective cancer cells. The cell-of-origin of OS is currently debatable. Both bone marrow-derived mesenchymal stem cells (BMSCs or MSCs) and osteoblasts have been presumed to be the cells-of-origin of OS [13–15].

RUNX2 is a lineage transcription factor for bone development. The 6p21 region that contains the RUNX2 gene is amplified in some osteosarcomas, in consistent with a role of RUNX2 in osteosarcomagenesis [16]. We and others have previously identified RUNX2 (Runx2 in mouse) as a p53-repressed target in OS and bone marrow-derived MSCs [17,18]. In this study, we explored the roles of RUNX2 in OS cells and our initial hypothesis was that RUNX2 knockdown delays the osteogenic differentiation of OS cells since RUNX2 inhibition delays osteogenic differentiation of MSCs. Unexpectedly, we found that RUNX2 depletion led to the apoptosis of OS cells. The loss of p53 enhances the apoptosis caused by RUNX2 depletion. Using integrative genome-wide approaches, we identified MYC as a novel target of RUNX2 in OS cells. Exogenously expressed MYC partially rescued apoptosis caused by RUNX2 knockdown. Furthermore, we found that RUNX2 recruits an epigenetic complex, the Menin/MLL1 complex, to facilitate the expression of MYC. Inhibition of the Menin/MLL complex using small-molecule inhibitors decreased the expression of MYC and induced apoptosis of p53 defective OS cells.

## Results

### RUNX2 depletion leads to apoptosis in p53 defective OS cells

**Fig 1:**
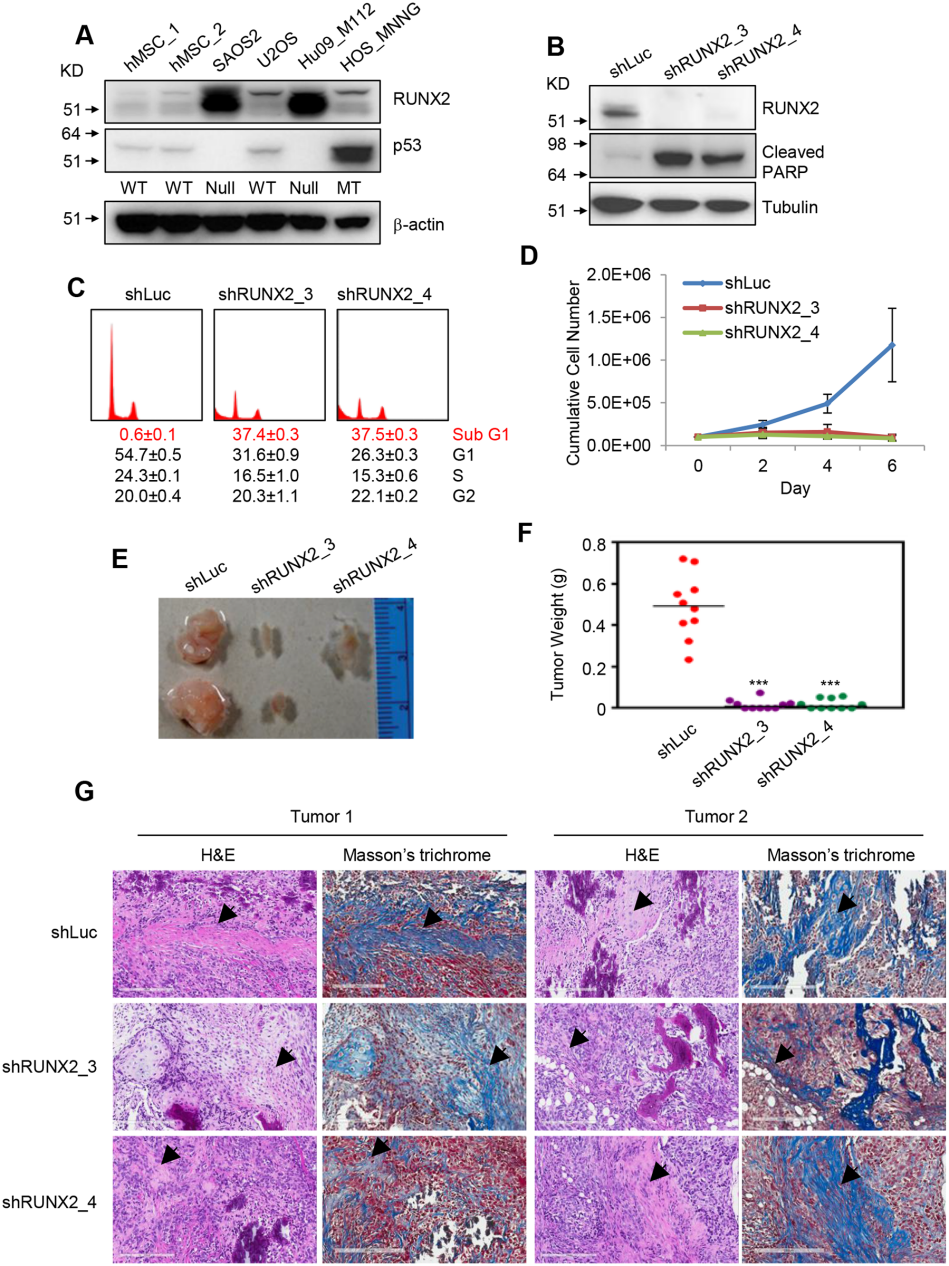
RUNX2 depletion causes apoptosis in OS cells. (**A**) Immunoblotting (I.B.) to examine the levels of RUNX2, p53 and β-actin in hMSCs and human OS cells. Below p53 I.B. are the genotypes of p53 of each cell line. WT, wild type; Null, p53 deletion; MT, mutant. (**B**) I.B. of RUNX2, cleaved PARP and Tubulin in SAOS2 cells transduced with lentiviruses expressing shLuc, shRUNX2_3 or shRUNX2_4. (**C**) Propidium iodide staining showing sub-G1 (apoptosis), G1, S, and G2 phases of SAOS2 cells transduced with lentiviruses expressing shLuc, shRUNX2_3 or shRUNX2_4. Numbers below histograms are percentages of phases. (**D**) Cumulative cell number of SAOS2 cells transduced with lentiviruses expressing shLuc, shRUNX2_3 or shRUNX2_4. (**E**) A representative image of tumors generated from SAOS2 cells transduced with lentiviruses expressing shLuc, shRUNX2_3 or shRUNX2_4. (**F**) Weight of tumors generated from SAOS2 cells transduced with lentiviruses expressing shLuc, shRUNX2_3 or shRUNX2_4 in NSG mice. ***, p<0.001; n = 10. (**G**) Hematoxylin and Eosin staining and Masson’s trichrome staining of SAOS2 tumors. Arrows indicate osteoid formation, the characteristics of OS.

To examine the levels of RUNX2 in OS cells, we performed immunoblotting (I.B.) analysis of RUNX2 in four different human OS cell lines and primary human mesenchymal stem cell (hMSC) from two individuals (Fig 1A).

SAOS2 and Hu09-M112 cells are p53 null, U2OS cells are p53 wild type, and HOS-MNNG cells carry a R156P p53 mutation [19]. RUNX2 levels were high in SAOS2 and Hu09-M112 cells and low in U2OS, HOS-MNNG cells, and hMSCs (Fig 1A).

We then investigated the effect of RUNX2 knockdown on OS cells. We chose SAOS2 and Hu09-M112 cells for our initial study because they express high levels of RUNX2 and undetectable levels of p53 protein (Fig 1A). In contrast to our initial hypothesis that RUNX2 affects OS cell differentiation, we found that RUNX2 knockdown led to apoptosis of SAOS2 cells based on cleaved PARP and propidium iodide staining (Fig 1B and 1C). SAOS2 cells with RUNX2 knockdown eventually died after several passages (Fig 1D). We observed similar results in Hu09-M112, whereby RUNX2 knockdown increased apoptosis (S1A–S1D Fig).

RUNX2 depletion significantly decreased the size of tumors generated from xenografts of SAOS2 cells in immunocompromised NSG mice (Fig 1E and 1F), suggesting that RUNX2 is also required for the survival of OS cells *in vivo*. Tumors in the absence or presence of RUNX2 knockdown have OS characteristics, such as osteoid formation (Fig 1G). Therefore, we concluded that RUNX2 is required for the survival of p53 null OS cells.

### RUNX2 depletion causes apoptosis of OS cells irrespective of p53 status

**Fig 2:**
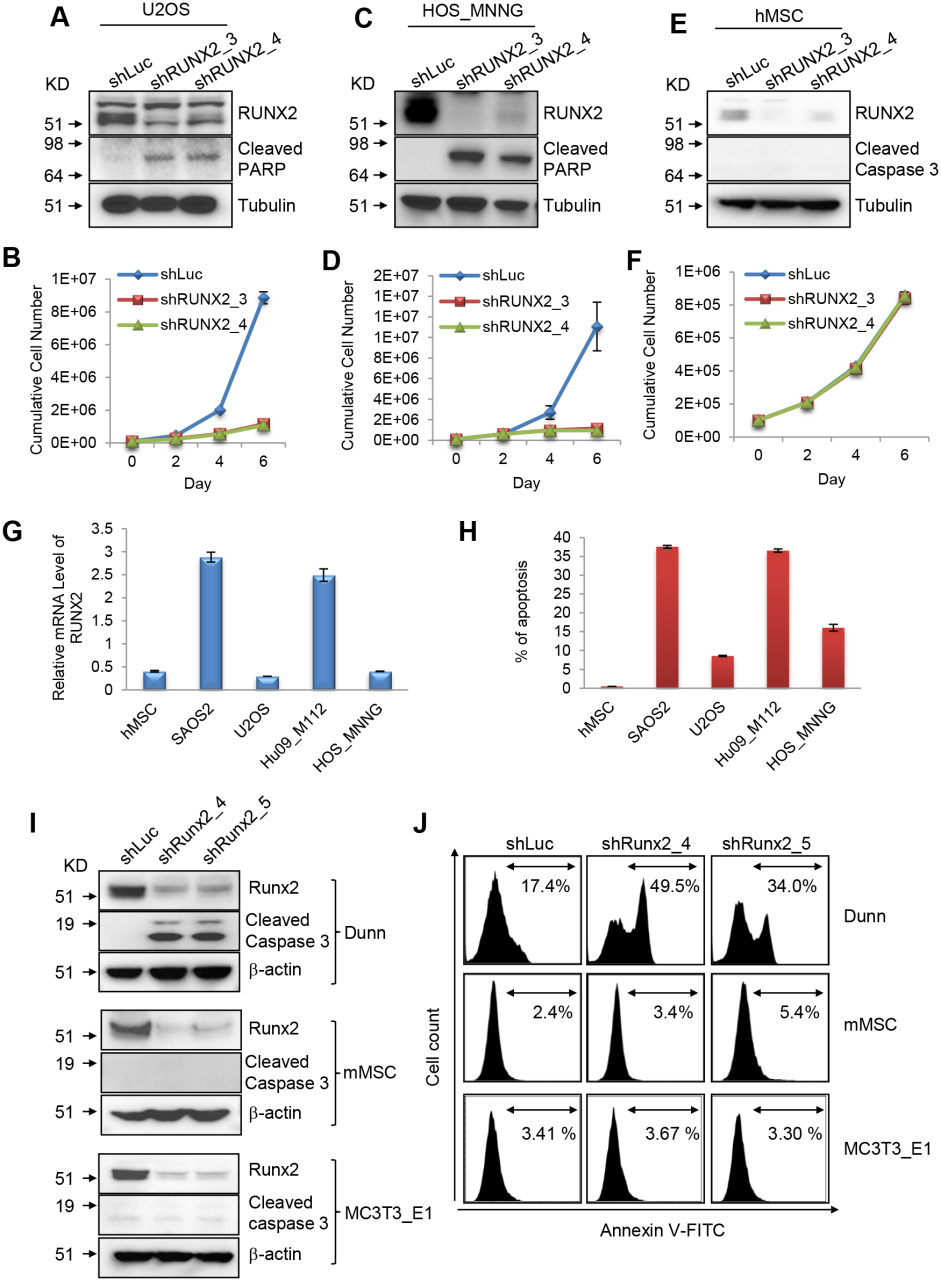
RUNX2 has a pro-survival function in OS cells but not in MSCs. (**A**) I.B. to detect RUNX2, cleaved PARP and Tubulin in U2OS cells transduced with lentiviruses expressing shLuc, shRUNX2_3 or shRUNX2_4. (**B**) Cumulative cell numbers of U2OS cells transduced with lentiviruses expressing shLuc, shRUNX2_3 or shRUNX2_4. (**C**) I.B. to detect RUNX2, cleaved PARP and Tubulin in HOS-MNNG cells transduced with lentiviruses expressing shLuc, shRUNX2_3 or shRUNX2_4. (**D**) Cumulative cell numbers of HOS-MNNG cells transduced with lentiviruses expressing shLuc, shRUNX2_3 or shRUNX2_4. (**E**) I.B. to detect RUNX2, cleaved caspase3 and Tubulin in hMSC cells transduced with lentiviruses expressing shLuc, shRUNX2_3 or shRUNX2_4. (**F**) Cumulative cell numbers of hMSC cells transduced with lentiviruses expressing shLuc, shRUNX2_3 or shRUNX2_4. (**G**) mRNA levels of RUNX2 in hMSCs and four different human OS cell lines. (**H**) Percentage of apoptotic cells of hMSCs and four different human OS cell lines 6 days after transduced with lentiviruses expressing 50/50 of shRUNX2_3 and shRUNX2_4. (**I**) I.B. to detect Runx2, cleaved caspase 3, β-actin in Dunn (mouse OS cells), mMSC, and MC3T3_E1 cells transduced with lentiviruses expressing shLuc, shRunx2_4 or shRunx2_5. (**J**) Annexin V staining of Dunn (mouse OS cells), mMSC, and MC3T3_E1 cells transduced with lentiviruses expressing shLuc, shRunx2_4 or shRunx2_5 for 4 days.

Since both SAOS2 and Hu09-M112 are p53 null, one possibility is that the loss of p53 reprograms these cells to depend on RUNX2 to survive. To test whether the apoptosis caused by RUNX2 knockdown requires p53 loss, we performed RUNX2 knockdown in U2OS (p53 wild type) and HOS-MNNG (p53R156P mutant) cells. RUNX2 knockdown caused apoptosis in both U2OS (Fig 2A and 2B and S1E Fig) and HOS-MNNG cells (Fig 2C and 2D and S1F Fig).

Thus, RUNX2 depletion leads to apoptosis of OS cells irrespective of p53 status.

To examine whether the pro-survival function of RUNX2 exists in hMSCs, we reduced the level of RUNX2 in hMSCs (Fig 2E). RUNX2 knockdown did not have any effects on apoptosis or proliferation of hMSCs (Fig 2E and 2F and S1G Fig). hMSCs had slightly lower levels of RUNX2 as compared to U2OS and HOS-MNNG cells, suggesting that the levels of RUNX2 cannot fully explain the pro-survival function of RUNX2 in OS cells.

We then addressed whether the levels of RUNX2 correlate with the degree of apoptosis of OS cells. The protein (Fig 1A) and mRNA levels (Fig 2G) of RUNX2 were correlated with the degree of apoptosis in OS cells (Fig 2H). RUNX2 knockdown had a more prominent effect on apoptosis in SAOS2 and Hu09-M112 cells than in U2OS and HOS-MNNG cells.

### The pro-survival function of Runx2 is conserved in mouse OS cells

To investigate whether the pro-survival function of Runx2 is conserved in mouse OS cells, we reduced the levels of Runx2 in Dunn cells (a mouse OS cell line), mouse MSCs (mMSCs) and MC3T3_E1 cells (a mouse pre-osteoblast cell line) by knockdown. Runx2 depletion only caused apoptosis in Dunn cells but not in non-transformed mMSCs and MC3T3_E1 (Fig 2I and 2J), suggesting that the pro-survival function of Runx2 is conserved in mouse OS cells. Along with the observation in human OS cells and hMSCs, it appears that RUNX2 acquires a conserved pro-survival function in OS cells while this function is dispensable for the survival of mMSCs and pre-osteoblasts.

### The loss of p53 enhances apoptosis caused by RUNX2 knockdown

**Fig 3:**
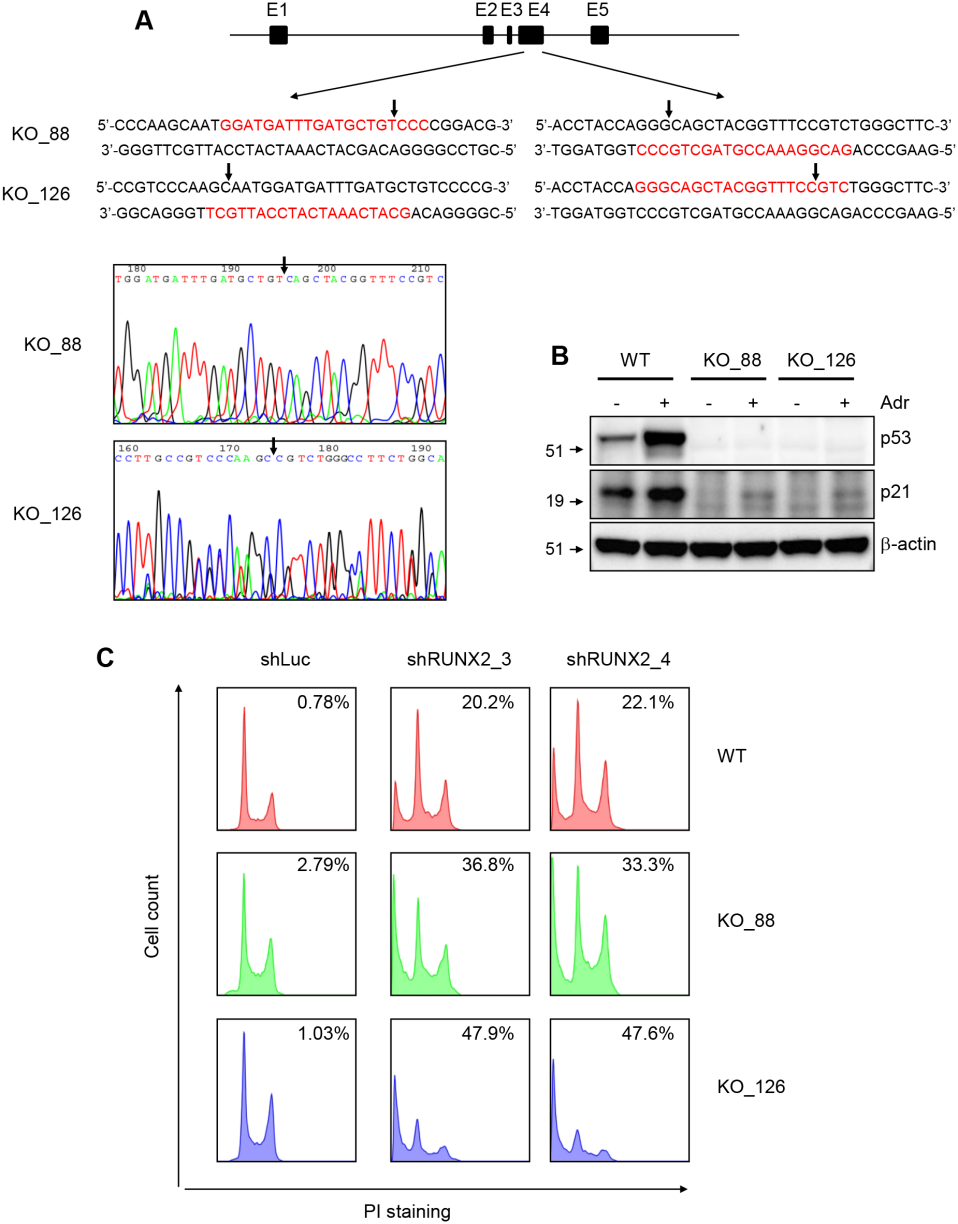
The loss of p53 enhances apoptosis caused by Runx2 knockdown. (**A**) Schematics showing the strategy of generating p53 knockout in U2OS cells. E1–E4, Exon1-Exon4 of the p53 gene. CRISPR targeting sequences are highlighted in red. Arrows indicate Cas9 cutting positions in the genome and ligation positions in chromatograms. KO-126 clone has an extra C (cytosine), which does not affect the knockout of p53 (see **B** for W.B.). (**B**) W.B. showing the loss of p53 protein and function in p53 KO clones. (**C**) Propidium iodide staining showing that p53 loss enhances apoptosis caused by RUNX2 knockdown. Numbers in each histogram show the percentage of sub-G1 phase.

Our results showed that p53 is not required for RUNX2 depletion-caused apoptosis, since RUNX2 knockdown caused apoptosis in p53 null SAOS2 and Hu09-M112 cells. However, it is unclear whether p53 loss has an effect on the degree of apoptosis in cells expressing wild type p53. Although a higher degree of apoptosis was observed in p53 null SAOS2 and Hu09-M112 cells than in p53 wild type U2OS cells, the difference could result from genetic difference of the individuals from which these cells are derived. To overcome this genetic background issue, we used CRISPR (clustered regularly interspaced short palindromic repeats) technology to generate p53 knockout (KO) isogenic cell lines from U2OS cells (Fig 3A) [20].

We used two different pairs of CRISPR constructs to delete part of the Exon 4 of the p53 mRNA (Fig 3A), and the deletions caused a loss of p53 protein in U2OS cells (Fig 3B). Using p21 as a functional readout, we confirmed that these clones (KO_88 and KO_126) have loss of function of p53 (Fig 3B). We then performed RUNX2 knockdown in these p53 knockout clones as well as the parental clone (p53_WT) and found that the loss of p53 enhances the apoptosis induced by RUNX2 knockdown (Fig 3C).

### CBFB, the binding partner of RUNX2, is up-regulated in and required for the survival of OS cells

**Fig 4:**
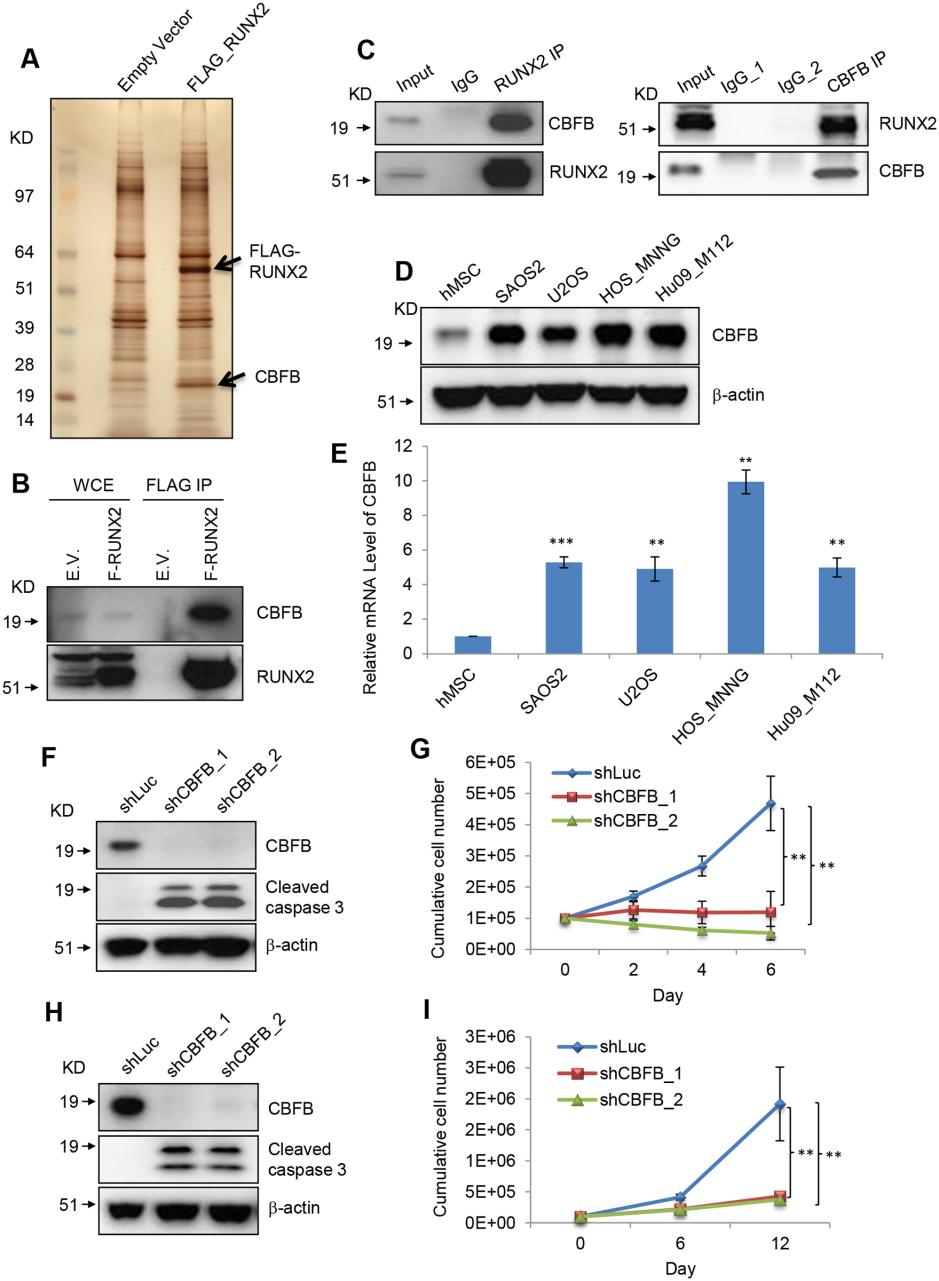
RUNX2 binding partner, CBFB, is required for OS cell survival. (**A**) Silver staining of FLAG-RUNX2 pulldown in SAOS cells. (**B**) I.B. to validate the interaction between CBFB and RUNX2 in SAOS2 cells. (**C**) Endogenous co-immunoprecipitation (Co-IP) assay of RUNX2 and CBFB in SAOS2 cells. (**D**) I.B. to detect CBFB in hMSCs and OS cells. (**E**) Realtime PCR to detect RNA levels of CBFB in hMSCs and OS cells. (**F**) I.B. to detect CBFB, cleaved caspase 3, and β-actin in SAOS2 cells transduced with lentiviruses expressing shLuc, shCBFB_1 or shCBFB_2. (**G**) Cumulative cell number of SAOS2 cells transduced with lentiviruses expressing shLuc, shCBFB_1 or shCBFB_2. (**H**) I.B. to detect CBFB, cleaved caspase 3, and β-actin in Hu09-M112 cells transduced with lentiviruses expressing shLuc, shCBFB_1 or shCBFB_2. (**I**) Cumulative cell number of Hu09-M112 cells transduced with lentiviruses expressing shLuc, shCBFB_1 or shCBFB_2. Error bars are SEM; t-test, **, p<0.01; *, p<0.05.

To explore the molecular mechanisms underlying the *de novo* pro-survival function of RUNX2 in OS cells, we set out to test the possibility that RUNX2 acquires an OS cell-specific binding partner that confers the pro-survival function on RUNX2. For this, we established a SAOS2 cell line that stably expresses FLAG-tagged RUNX2 (Fig 4A) and performed FLAG IP followed by mass spectrometry to detect the proteins enriched in FLAG-RUNX2 IP.

We identified CBFB (core binding factor beta) as a binding partner of RUNX2 in SAOS2 cells (Fig 4A and 4B) and Hu09-M112 cells (S2A and S2B Fig). Reciprocal co-IP experiments showed that endogenous RUNX2 and CBFB interact in OS cells (Fig 4C). CBFB has previously been shown to be a *bona fide* binding partner of RUNX2 in osteoblasts and to enhance the binding of RUNX2 to DNA [21]. Therefore, CBFB binding to RUNX2 in OS cells cannot explain the *de novo* survival function of RUNX2 in OS cells. Western blot analysis showed that CBFB is up-regulated in OS cells compared to hMSCs (Fig 4D).

Realtime PCR showed that the mRNA levels of CBFB were higher in OS cells compared to hMSCs (Fig 4E). The mechanism underlying the up-regulation of CBFB in OS cells is currently unknown. The degree of the up-regulation does not appear to be p53-dependent.

We then tested whether CBFB is also required for the survival of OS cells and found that CBFB depletion in SAOS2 and Hu09-M112 cells gave rise to apoptosis in these two cell lines (Fig 4F–4I). However, CBFB knockdown in hMSCs did not lead to apoptosis (S2C and S2D Fig). Therefore, CBFB knockdown has the same outcome as RUNX2 knockdown. Since CBFB and RUNX2 both are involved in transcriptional regulation, these results suggest the downstream transcriptional targets of RUNX2/CBFB complex mediate the pro-survival function of RUNX2.

### RUNX2 and CBFB binding sites are highly overlapping

**Fig 5:**
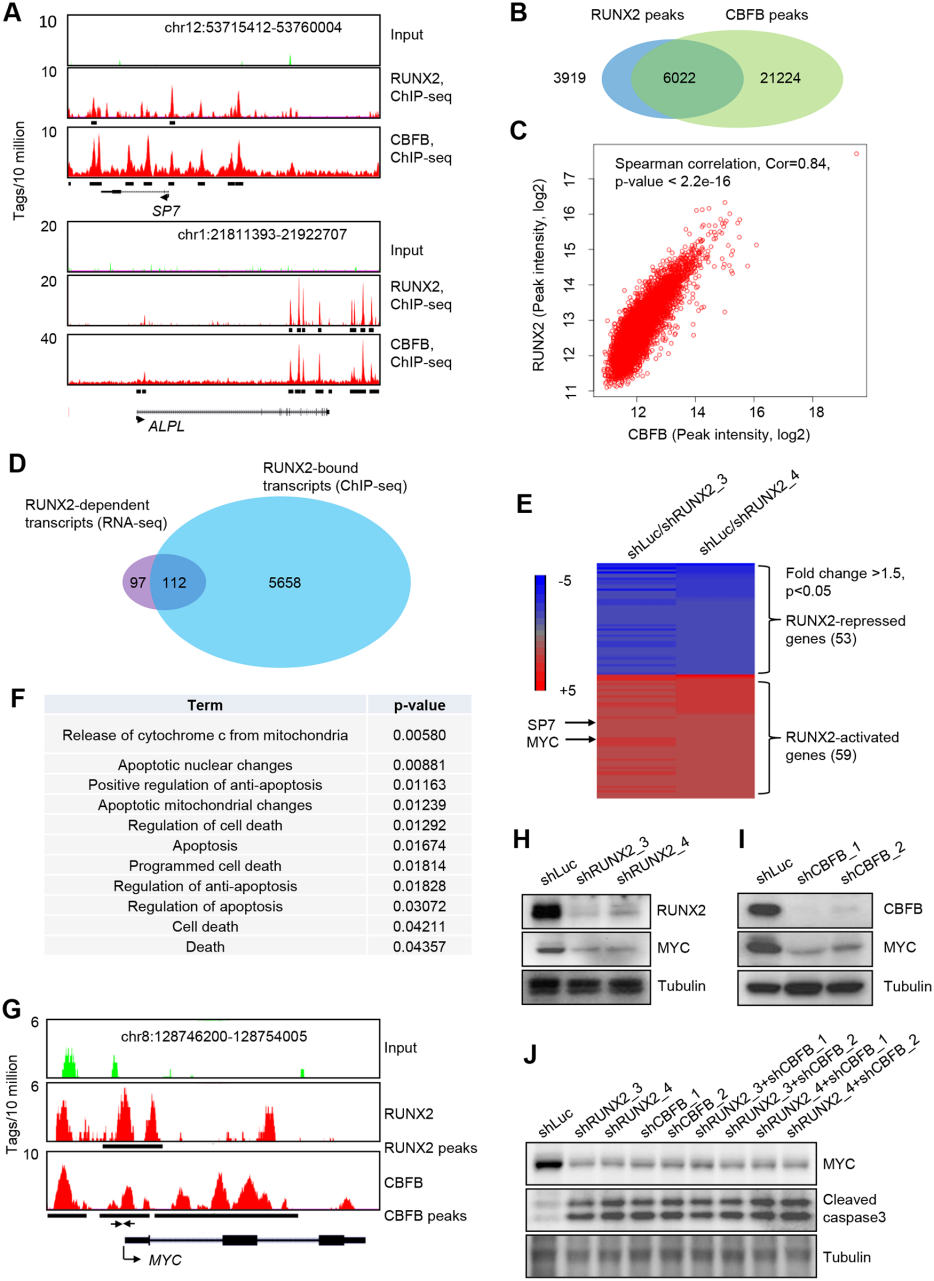
RUNX2 and CBFB co-occupy many loci in the genome. (**A**) Genomic views of RUNX2 and CBFB binding on the *SP7* and *ALPL* loci. Black bars underneath genomic view are identified peaks by the MACS algorithm. (**B**) Venn diagram of the numbers of RUNX2 peaks and CBFB peaks. (**C**) Spearman correlation of peaks intensity of 6022 overlapping RUNX2 and CBFB peaks. See Methods for calculation of peak intensity. (**D**) Venn diagram of RUNX2-dependent transcripts and RUNX2-bound transcripts. (**E**) Heatmap showing expression changes of the 112 RUNX2 direct targets (fold change >1.5, p<0.05) in SAOS2 cells. Showing is the average of three repeats from RNA-seq. See Methods for integration of RNA-seq and ChIP-seq. (**F**) Gene Ontology (GO) terms of genes enriched in the 112 RUNX2 direct targets. (**G**) Genomic view of RUNX2 and CBFB binding on the MYC locus. The black bars underneath represents identified peaks using the MACS algorithm. (**H**) I.B. to detect RUNX2, MYC and Tubulin in SAOS2 cells transduced with lentiviruses expressing shLuc, shRUNX2_3 or shRUNX2_4. (**I**) I.B. to detect CBFB, MYC and Tubulin in SAOS2 cells transduced with lentiviruses expressing shLuc, shCBFB_1 or shCBFB_2. (**J**) I.B. to detect MYC expression and apoptosis in SAOS2 cells with single or double knockdown of RUNX2 and CBFB.

To identify the downstream targets of RUNX2 that may be involved in the pro-survival function of RUNX2, we used an integrative genome-wide approach combining ChIP-seq (chromatin immunoprecipitation followed by deep sequencing) and RNA-seq [22]. We performed ChIP-seq of RUNX2 and CBFB in SAOS2 cells. Using two well-established targets of RUNX2, *SP7* (also known as *Osterix*) and *ALPL*, we found that the binding sites of RUNX2 and CBFB were highly overlapping at these two loci (Fig 5A).

CBFB does not directly bind to DNA. Instead, it hetero-dimerizes with the members (RUNX1, RUNX2 and RUNX3) in the RUNX family and enhances their DNA binding [23,24]. At the genome-wide level, 61% (6022 out of 9941) of RUNX2 peaks overlapped with CBFB peaks (Fig 5B). Peak intensities (binding strength) of the 6022 overlapping peaks of RUNX2 and CBFB are highly correlated (Spearman correlation, cor = 0.84, p<2.2e-16), supporting the notion that CBFB facilitates the DNA binding of RUNX2 (Fig 5C). We identified more CBFB peaks than RUNX2 peaks presumably due to the fact that CBFB may act as a transcriptional cofactor for other transcription factors [24,25]. The high degree of overlapping of RUNX2 and CBFB binding suggests that the transcriptional regulation by RUNX2/CBFB mediates the pro-survival function of RUNX2.

### MYC is a direct target of RUNX2 and CBFB

After obtaining the high quality ChIP-seq dataset for RUNX2, we performed RNA-seq analyses of SAOS2 cells transduced with shLuc, shRUNX2_3 or shRUNX2_4. We selected only those transcripts that were changed in the same direction with both shRNAs to minimize off-target effects. These analyses resulted in 209 RUNX2-dependent genes (S1 Table). We assigned RUNX2 peaks from ChIP-seq to specific genes and identified 5770 genes that have at least one RUNX2 binding site less than 25 kb away (S2 Table, also see Methods). Because the number of targets bound by RUNX2 is much greater than that of targets with RUNX2-dependent changes in expression (Fig 5D), only a small fraction of RUNX2 binding sites is functional in transcription.

We then integrated RNA-seq data with the ChIP-seq dataset to identify RUNX2 direct targets (Fig 5D), which are defined as those with a RUNX2-dependent change in gene expression (measured by RNA-seq) and having at least one associated RUNX2 peak (measured by ChIP-seq). This integration resulted in 112 RUNX2 direct targets: 53 RUNX2-repressed and 59 RUNX2-activated (Fig 5E). To narrow down the critical mediator of the pro-survival function of RUNX2, we searched for enriched pathways and focused on those related to apoptosis or cell death. A total of 11 apoptosis-related pathways were enriched in RUNX2 direct targets (Fig 5F). We then interrogated the genes within the 11 enriched apoptosis-related pathways and found that MYC is one of the commonly shared genes among the pathways. MYC is known to play important roles in proliferation and survival of many cancer cells [26]. Both RUNX2 and CBFB bound to the promoter of the MYC gene (Fig 5G). Knockdown of RUNX2 and CBFB decreased the expression of the MYC gene in SAOS2 cells (Fig 5H and 5I) and Hu09-M112 cells (S3 Fig), further validating our finding that MYC is a downstream target of the RUNX2/CBFB complex in OS cells. Double knockdown of RUNX2 and CBFB in SAOS2 cells still did not lead to a complete loss of MYC expression (Fig 5J), probably due to the incomplete loss of RUNX2 and CBFB in the knockdown or the existence of other regulatory mechanisms of MYC expression.

### MYC is one of the mediators of the pro-survival function of RUNX2 and CBFB

**Fig 6:**
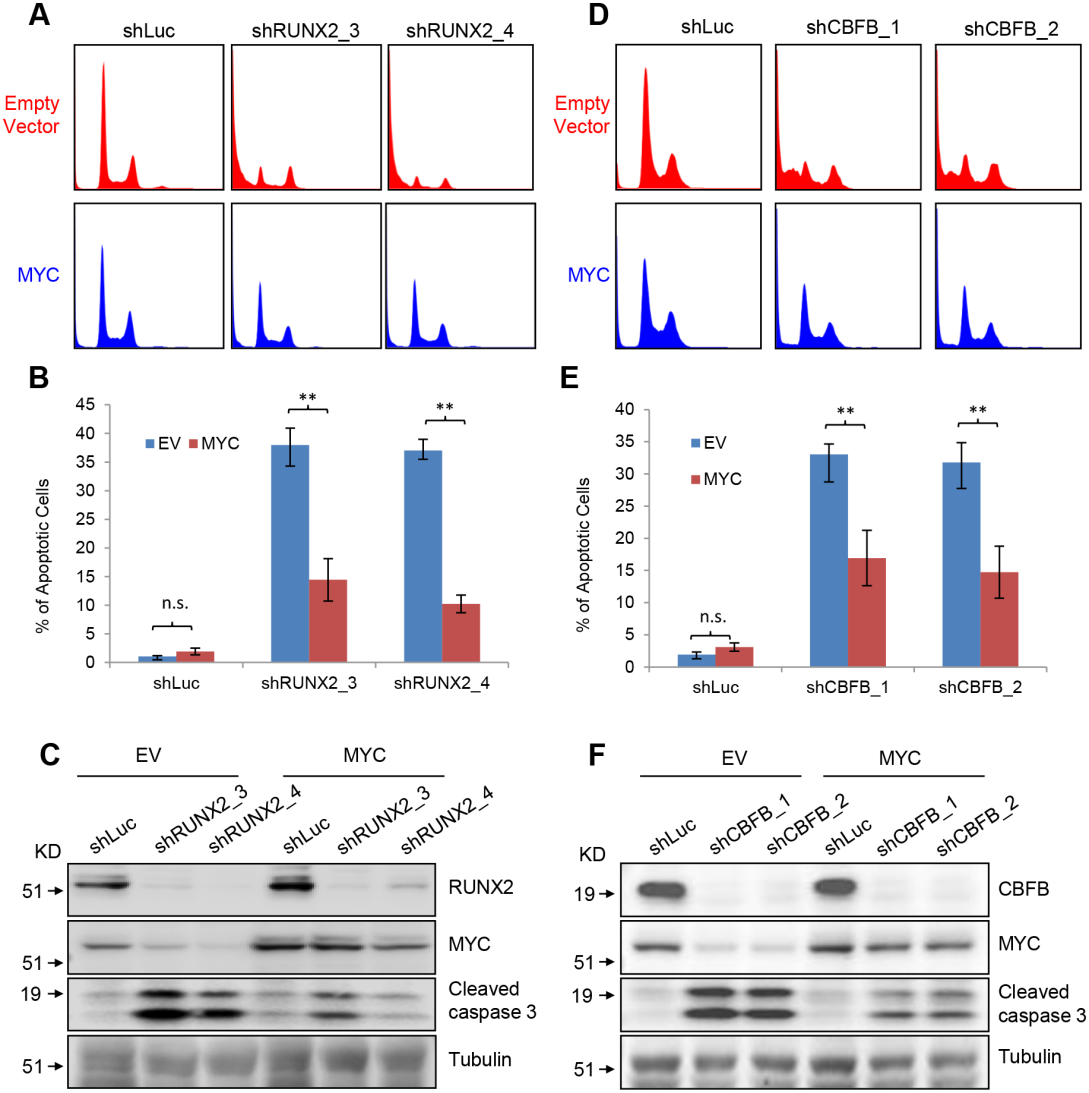
Exogenously expressed MYC partially rescues apoptosis caused by RUNX2 or CBFB knockdown. (**A**) Histogram of propidium iodide staining of rescue experiments in SAOS2 cells transduced with empty vector or MYC vector followed by transduction with shLuc, shRUNX2_3 or shRUNX2_4 lentiviruses. (**B**) Quantitative analyses of sub-G1 phase of the rescue experiments in (**A**). Error bars are SEM; t-test, **, p<0.01; *, p<0.05. (**C**) I.B. to detect RUNX2, MYC, cleaved caspase 3, and Tubulin in the rescue experiments in SAOS2 cells transduced with lentiviruses expressing shLuc, shRUNX2_3 or shRUNX2_4. (**D**) Histogram of propidium iodide staining of rescue experiments in SAOS2 cells transduced with empty vector or MYC vector followed by transduction with lentiviruses expressing shLuc, shCBFB_1 or shCBFB_2. (**E**) Quantitative analyses of sub-G1 phase of the rescue experiments in (**D**). Error bars are SEM; t-test, **, p<0.01; *, p<0.05. (**F**) I.B. to detect CBFB, MYC, cleaved caspase 3, and Tubulin in the rescue experiments in SAOS2 cells transduced with lentiviruses expressing shLuc, shCBFB_1 or shCBFB_2.

To test whether MYC mediates the pro-survival function of RUNX2 and CBFB, we established SAOS2 cells that stably carry either an empty vector or a MYC-expressing vector. We then reduced RUNX2 levels these SAOS2 cells by knockdown. Exogenously expressed MYC significantly decreased apoptosis caused by RUNX2 depletion (Fig 6A–6C).

The decreased apoptosis was also associated with increased cumulative cell number (S4A Fig). We also found that exogenous MYC expression rescued CBFB knockdown (Fig 6D–6F and S4B Fig), supporting the notion that MYC is a mediator of the pro-survival function of the RUNX2/CBFB complex in OS cells. Since exogenous MYC only partially rescues the apoptosis caused by RUNX2 or CBFB knockdown (Fig 6B and 6E), other factors exist to mediate the pro-survival function of the RUNX2/CBFB complex in OS cells.

### MYC is up-regulated in OS tumors and is required for the survival of OS cells

**Fig 7:**
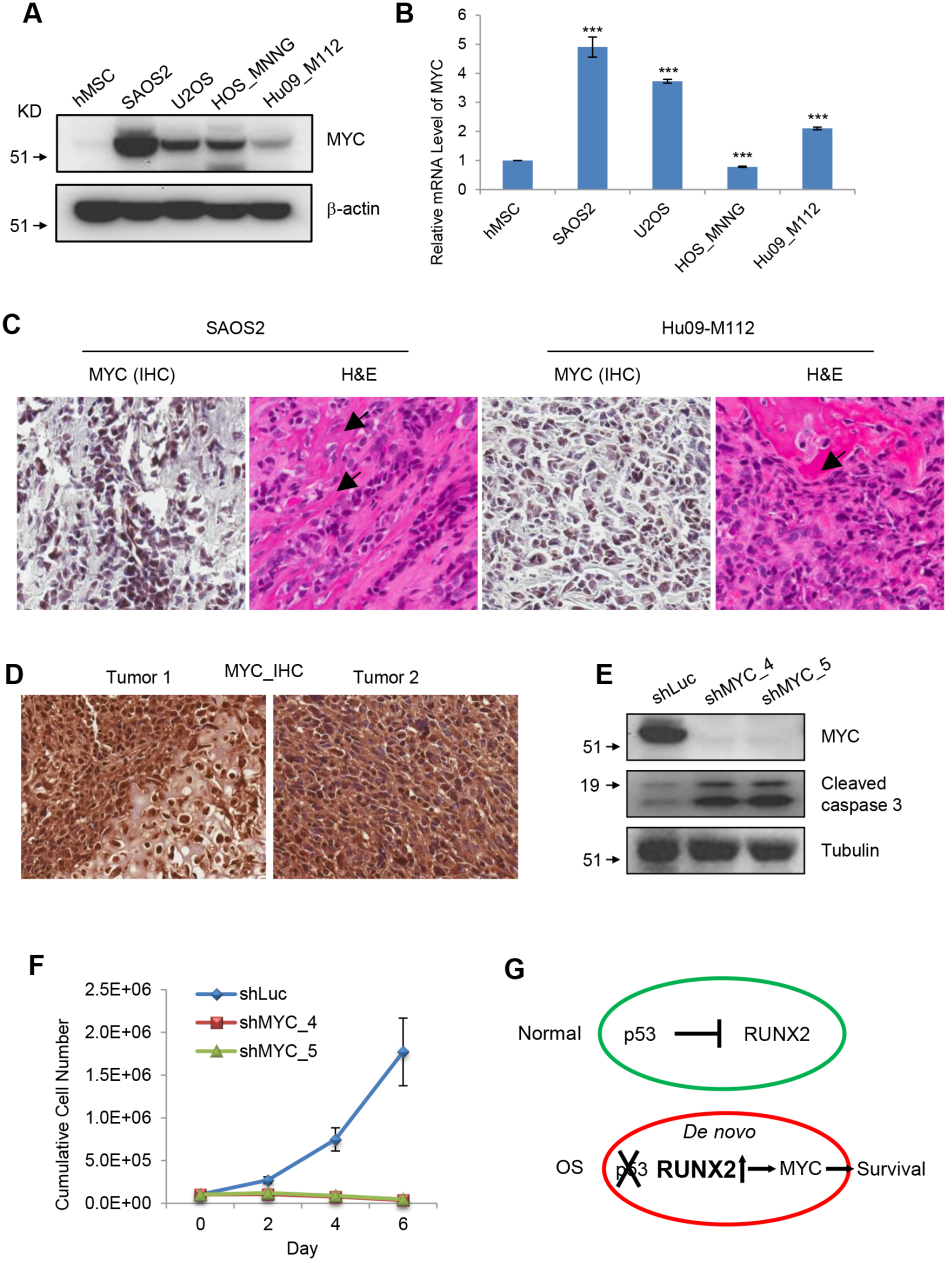
MYC is up-regulated in and required for the survival of OS cells. (**A**) I.B. to detect MYC and β-actin in hMSCs and OS cells. (**B**) Realtime PCR to measure the levels of MYC transcript in hMSCs and OS cells. (**C**) MYC immunohistochemistry (IHC) and H&E staining of xenografted SAOS2 and Hu09-M112 tumors. Arrows indicate osteoid formation. (**D**) MYC IHC in OS patient tumors. (**E**) I.B. of MYC, cleaved caspase 3, and Tubulin in SAOS2 cells. (**F**) Cumulative cell numbers of SAOS2 cells transduced with lentiviruses expressing shLuc, shMYC_4 or shMYC_5. (**G**) A model of RUNX2-MYC in p53-independent apoptosis of OS cells.

To examine the expression levels of MYC in OS cells, we performed immunoblotting of MYC in hMSCs and OS cells and found that MYC is up-regulated in OS cells compared to hMSCs (Fig 7A).

The mRNA of MYC was also significantly higher in OS cells compared to hMSCs with the exception of the HOS-MNNG OS cell line (Fig 7B). We also observed that Myc is up-regulated in mouse OS cells (S5A Fig), suggesting that up-regulation of MYC (Myc) is a conserved phenomenon in OS cells. MYC positive staining was also readily observed in the xenografts of SAOS2 and Hu09-M112 cells in NSG mice (Fig 7C) and human OS patient samples (Fig 7D and S5B Fig). Knockdown of MYC in SAOS2 cells increased apoptosis (Fig 7E) and decreased the cumulative cell number (Fig 7F). Similar results were observed in Hu09-M112 cells (S5C and S5D Fig). Together, our results revealed that MYC is up-regulated by RUNX2 and required for the survival of OS cells (Fig 7G).

### A RUNX2-mediated epigenetic mechanism that regulates MYC expression

**Fig 8:**
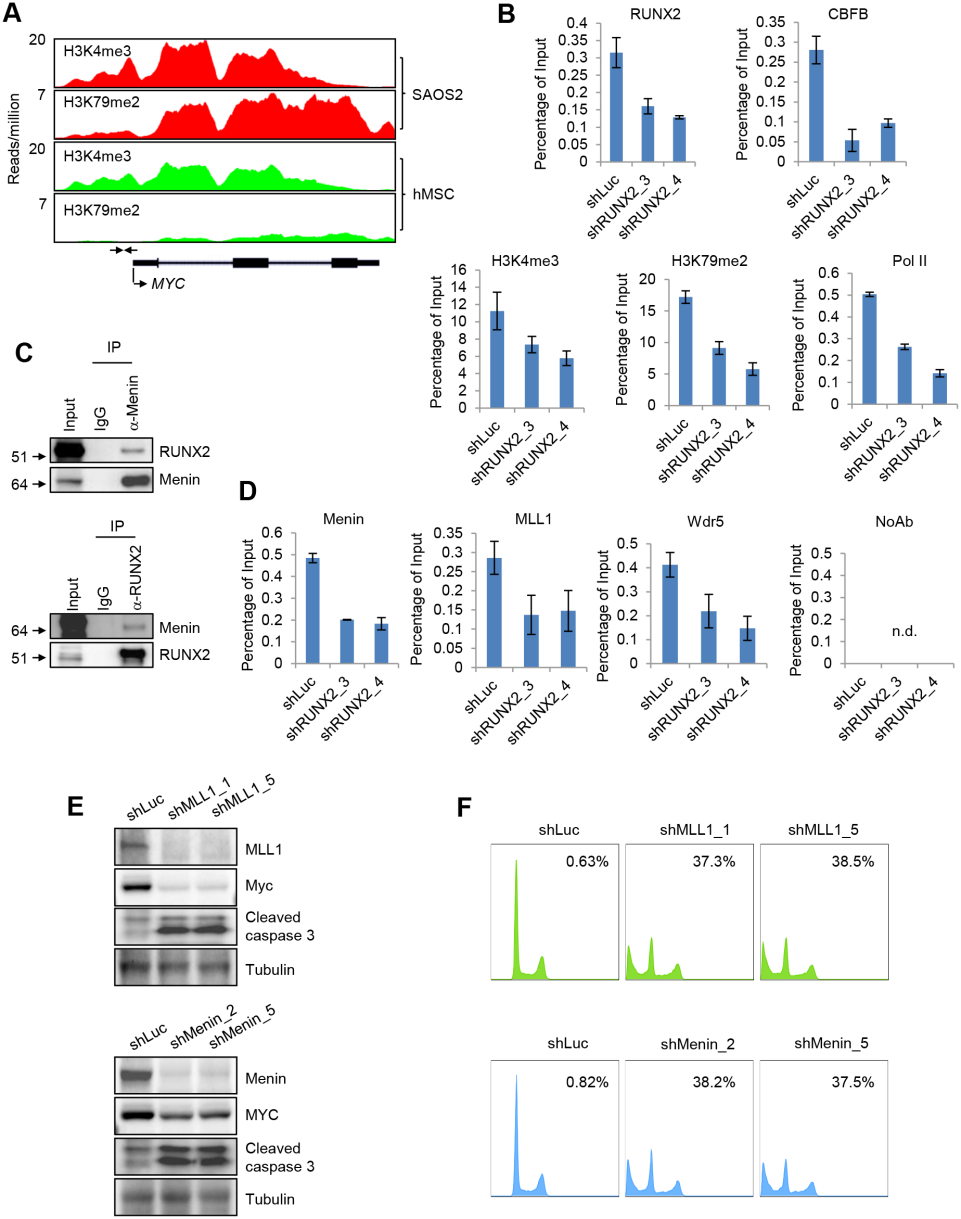
A RUNX2-mediated epigenetic mechanism that regulates MYC expression in OS cells. (**A**) A genomic view of H3K4me3 and H3K79me2 ChIP-seq on the MYC locus in SAOS2 cells and hMSCs. The two head-to-head arrows indicate the ChIP amplicon region, which is the RUNX2 binding peak shown in Fig 5G. (**B**) ChIP assay to assess the effect of RUNX2 knockdown on the levels of CBFB, H3K4me3, H3K79me2, and Pol II on the MYC promoter in SAOS2 cells. The amplicon region was indicated in (**A**). (**C**) RUNX2 and Menin interact in SAOS cells. (**D**) ChIP assay to assess the effect of RUNX2 knockdown on the levels of Menin, MLL1, and Wdr5 recruitment on the MYC promoter. NoAb, no antibody control. (**E**) Immunoblotting and (**F**) flow cytometry showing that MLL1 (upper panels) and Menin (lower panels) knockdown led to the apoptosis of SAOS2 cells.

We explored a possible epigenetic and/or transcriptional regulation of MYC transcription in OS cells since genome-wide sequencing studies did not identify any consistent oncogenes in OS [7,8]. Using ChIP-seq, we mapped histone H3 lysine 4 trimethylation (H3K4me3), a transcription initiation mark, and H3 lysine 79 dimethylation (H3K79me2), a transcription elongation mark. We found that H3K4me3 and H3K79me2 levels are high in SAOS2 cells compared to hMSCs, supporting an epigenetic and/or transcriptional mechanism for the up-regulation of the MYC gene (Fig 8A).

Because RUNX2 regulated the expression of MYC, we investigated whether RUNX2 is involved in the up-regulation of these two histone modifications. We found that RUNX2 knockdown decreased the levels of these two modifications as well as RNA polymerase II (Pol II) (Fig 8B). Complexes that carry out H3K4me3 are the COMPASS-like complexes, which consist of MLL1, Menin and other subunits [27]. Interestingly, both MLL1 and Menin, like RUNX2, are involved in the normal physiology of skeletal system and osteocyte maturation [28,29]. Although previous studies showed that the COMPASS-like complex suppresses leukemogenesis, the tumor promoting role of this complex in solid tumors, such as breast tumors, has been noted [30], suggesting a contextual role of this complex in cancer. Since Menin is the scaffold protein in the COMPASS complex, we tested the interaction between RUNX2 and Menin and detected interaction between these two proteins at endogenous levels (Fig 8C). To further test whether MLL1 and Menin directly target MYC expression, we performed ChIP assays (Fig 8D). Menin, MLL1, and another subunit Wdr5 were recruited to the MYC promoter (Fig 8D). Importantly, RUNX2 knockdown decreased the recruitment of these proteins (Fig 8D), suggesting that the recruitment of the COMPASS-like complex to the MYC promoter is regulated by RUNX2. Similar to RUNX2 knockdown, MLL1 and Menin knockdown decreased the expression of MYC and increased apoptosis (Fig 8E and 8F).

**Fig 9:**
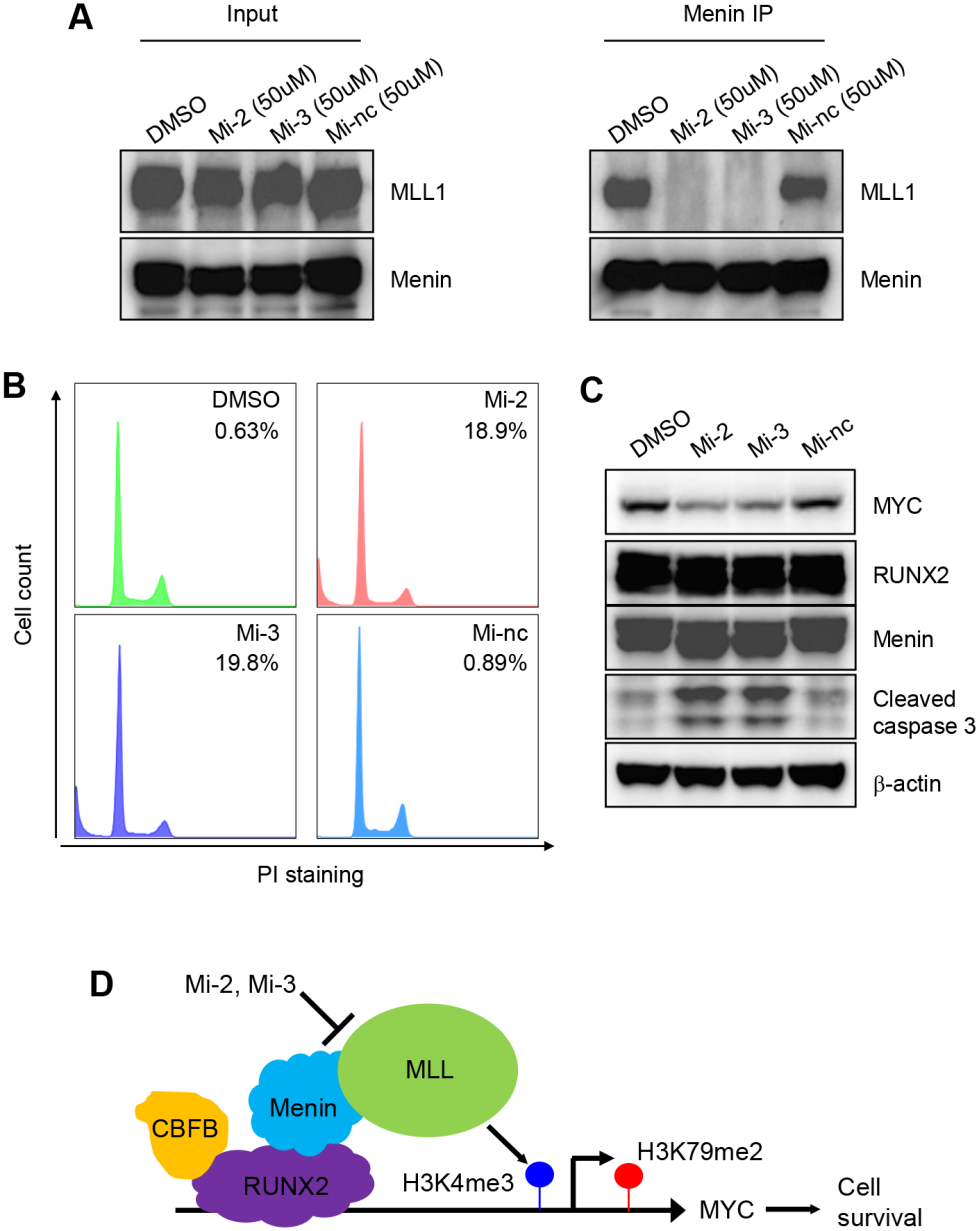
Menin inhibitors induce the apoptosis in OS cells. (**A**) Immunoprecipitation (IP) showing the effect of Mi-2, Mi-3, and Mi-nc on the interaction between Menin and MLL1. (**B**) Flow cytometry and (**C**) W.B. showing that Mi-2 and Mi-3 induce the apoptosis of SAOS2 cells. (**D**) A model of a RUNX2-mediated epigenetic mechanism that regulates MYC expression and the survival of OS cells.

The interaction of Menin and MLL1 is required for the activity of MLL1 [31]. Recently, two inhibitors of disrupting the Menin/MLL1 interaction, Mi-2 and Mi-3 (Menin inhibitor 2 and 3), have been reported [32]. We decided to test whether these two inhibitors induce apoptosis in OS cells. As a control, we included an inactive inhibitor, Mi-nc. Co-IP experiments in SAOS2 cells showed that Mi-2 and Mi-3 decreased the interaction between Menin and MLL1 while Mi-nc did not (Fig 9A).

We found that Mi-2 and Mi-3 induced the apoptosis and decreased MYC expression in OS cells (Fig 9B and 9C), suggesting that pharmacological inhibition of Menin/MLL1 interaction induces apoptosis in OS cells. Although we cannot completely exclude the possibility that Mi-2 and Mi-3 affect the expression of other regulators of MYC expression, our results strongly support the notion that the Menin/MLL1 complex is involved in the regulation of MYC expression based on the recruitment of Menin and MLL1 to the MYC promoter (Fig 8D). Together, our data provide a proof-of-concept that the pharmacological inhibition of this epigenetic complex is a promising strategy of inducing apoptosis of OS cells (Fig 9D).

## Discussion

### Epigenetic regulation of the survival of OS cells

Sarcomas, on average, have less number of mutations compared to other types of tumors, suggesting that epigenetic mechanisms play important roles in their etiology [33]. Here, we studied the pro-survival function of RUNX2 in OS cells and found that RUNX2 induces the expression of MYC. Further, we discovered an epigenetic mechanism involving RUNX2, the Menin/MLL1 complexes, and MYC that regulates the survival of OS cells (Fig 9D). The Menin/MLL1 complexes have previously been shown to be tumor suppressive in blood cells as the mutations or translocations of MLL are associated with leukemogenesis [27]. In contrast, a recent study showed that these complexes could promote breast tumor growth, suggesting that the roles of the Menin/MLL1 complexes in cancer depend on cancer types [30]. Our result showed that these epigenetic complexes also are involved in the survival of OS cells. Using small-molecule inhibitors, Mi-2 and Mi-3, we demonstrated that the inhibition of the activities of these complex induced apoptosis of OS cells. Thus, our studies provide a proof-of-concept for killing OS by inhibiting the activity of an epigenetic complex.

### The p53-independent apoptosis of OS cells

The loss of p53 activity creates a major challenge for p53-related cancer therapy. To kill p53 deficient cancer cells, several context-dependent approaches were designed: if cancer cells have over-expressed MDM2, MDM2 inhibitors, such as Nutlin and RITA, can be used to activate p53 [3,4]. If cancer cells contain mutant p53, compounds that convert mutant p53 into the wild type p53-like conformation can be used [5]. However, in cancer cells with p53 deletions, the benefit of these approaches, which requires the existence of either wild type p53 or mutant p53, is diminished. Therefore, new approaches are needed for inducing apoptosis in cancer cells with a p53 deletion. An alternative approach is to target the survival signals in cancer cells irrespective of p53 status (p53 wild type, null or mutants) [6]. Our results showed that RUNX2 signaling is one of the survival signals in OS cells irrespective of p53 status. Because SAOS2 cells were derived from a chemo-resistant OS tumor, our study also provides a much needed means of killing chemo-resistant OS tumor cells [34]. Interestingly, although we started with OS cells carrying p53 deletion, we found that RUNX2 is also required for the survival of OS cells carrying mutant p53 (HOS-MNNG) or even wild type p53 gene (U2OS). It is probably due to the fact that the p53 signaling pathway as a whole is dysregulated in tumor versus non-tumor cells. We did observe higher degree of apoptosis in p53 null OS cells (SAOS2 and Hu09-M112) than in p53 wild type (U2OS) and p53 mutant (HOS-MNNG) OS cells. We note that these cells are derived from different patients and therefore, the difference in apoptosis may be caused by the individual differences of the patients from which these cells are derived. Using CRISPR to generate isogenic p53 knockout cell lines from U2OS, we established that p53 loss does increase the degree of apoptosis caused by RUNX2 depletion. Because RUNX2 is not needed for the survival of hMSCs and pre-osteoblasts, the pro-survival function of RUNX2 signaling in OS cells serves as an unprecedented opportunity to induce p53-independent apoptosis in these tumor cells.

RUNX2 is a key lineage factor for osteogenic differentiation and its level needs to be tightly controlled [35]. We and others have shown that p53 indirectly represses RUNX2 in MSCs and OS cells [18]. Thus, when p53 signaling is dysregulated in OS cells, OS cells could “hijack” the pro-survival function of RUNX2 that is normally needed for osteogenic differentiation. MSCs develop into pre-osteoblasts, osteoblasts, and eventually mature osteocytes. It is possible that the RUNX2/CBFB signaling acquires the pro-survival function in a middle stage during this dynamic differentiation process. If this is true, it explains why RUNX2 depletion does not cause apoptosis in human and mouse MSCs, which represent the initial stage of osteogenic differentiation.

### The roles of RUNX2 in cell cycle regulation and survival

RUNX2 has been shown to have a negative role in cell cycle progression [36,37]. For example, Galindo et al. showed that when being over-expressed, RUNX2 has an anti-proliferative role in MC3T3 (pre-osteoblast) and C2C12 (myoblast) cells [36]. Interestingly, siRNA-mediated knockdown of RUNX2 has no effect on cell cycle progression in MC3T3 cells. This result is similar to our observation that shRNA-mediated knockdown of RUNX2 has no effect on the survival of hMSCs, mMSCs, and MC3T3 cells. San Martin et al. examined the cell cycle-associated regulation of RUNX2 and CBFB and found that cell cycle regulation of these two proteins becomes aberrant in OS cells [37]. We did attempt to express RUNX2 at a very high level in SAOS2 cells when we established the stable cell line. Under this condition, we fail to obtain stable cell lines, suggesting that a very high level of RUNX2 impairs cell proliferation or survival. We overcame this negative effect of RUNX2 by lowering the titer of lentiviruses and eventually established SAOS2 cells that stably express Flag-tagged RUNX2 (Fig 4A). This preliminary result agrees to the conclusion that RUNX2 can act as a negative regulator of cell cycle. It appears that the level of RUNX2 has to be high enough for RUNX2 to have a negative effect on cell cycle. In addition, as shown by San Martin et al., RUNX2 level is dysregulated in OS cells, which may have higher tolerance to the RUNX2 level than osteoblasts or other cell types. The majority of our experiments are knockdown. Under this condition, we observed that RUNX2 is required for the survival of OS cells as RUNX2 depletion leads to apoptosis. Therefore, the roles of RUNX2 in cell cycle regulation and survival are cell type- and context-dependent.

### The regulation of MYC by RUNX2

In this study, we discovered MYC as a novel transcriptional target of RUNX2. We found that MYC is over-expressed in both human and mouse OS cell lines compared to hMSCs. Because MYC is a survival factor, this RUNX2/MYC axis provides an explanation for the pro-survival function of RUNX2. Indeed, exogenously expressed MYC partially rescued the apoptosis caused by RUNX2 and CBFB knockdown, further supporting the notion that RUNX2 provides survival signals to OS cells partially through MYC. Since it is challenging to directly target MYC due to its unstructured conformation [38], RUNX2-mediated regulation of MYC expression offers a much needed framework for developing agents to suppress MYC in p53-defective OS cells. We note that besides RUNX2, other factors could also regulate the expression of MYC in OS. Nevertheless, RUNX2 is required for the maintenance of MYC expression as RUNX2 knockdown has a prominent role in the reduction of MYC expression in OS cells.

### MYC may be an oncogene in OS

Tumor suppressors of OS cells, such as p53 and RB, have been well documented by genetic approaches. The critical roles of these tumor suppressors in OS are underscored by the high frequency of p53 and RB mutations in OS reported by recent genome-wide sequencing studies [7,8]. However, oncogenes for OS are less well known. Our study suggests that MYC is one of the oncogenes in OS cells given that it is highly expressed in both human and mouse OS cells. Genetic alterations, such as mutations and translocations, of the MYC gene have been reported in genome-wide sequencing studies [7,8]. The low frequency of these genetic alterations suggests that the dysregulation of MYC in OS cells may involve transcriptional and/or epigenetic mechanisms. Indeed, a transcriptional hypothesis is supported by our observations that RUNX2 is required for transcription of MYC in OS cells. Importantly, depletion of RUNX2 decreased MYC expression and increased the apoptosis of OS cells. As targeting MYC is an actively pursued arena, our study merits future testing of effects of emerging MYC-targeting compounds in killing OS cells. Since the expression of RUNX2 is generally restricted to the skeletal system, an alternative strategy could be targeting the RUNX2/CBFB interaction, which might produce less unwanted effects than targeting the ubiquitously expressed MYC.

## Methods

### Cell culture

hMSCs were purchased from ATCC and grown in hMSC medium (ATCC). U2OS, SAOS2 and HOS-MNNG cells were purchased from ATCC and grown in DMEM+10% FBS+antibiotics. Hu09-M112 is a subclone (generous gift from Dr. Jun Yokota, Biology Division, National Cancer Center Research Institute, Japan) from Hu09 cells and grown in RPMI1640+10% FBS+antibiotics [39]. mMSCs were isolated from the bone marrow of p53 knockout mice, as previously described [18]. Dunn cells and MC3T3-E1 cells (ATCC) were grown in DMEM+10% FBS+antibiotics.

### Xenograft tumor

Ten million SAOS2 cells were transduced with lentivirus expressing shLuc, shRUNX2_3 or shRUNX2_4 for 24 hours. Cells were re-suspended in 100 ul of PBS buffer+25mM HEPES, and mixed with 50 ul Matrigel before being transplanted into hind limb muscle of NSG mice. 43 days after transplantation, tumors were harvested, weighed and fixed in 10% neutral buffered formalin for 16 hours before Hematoxylin and Eosin staining. For Hu09-M112 cells, 1 million cells were transplanted into NSG mice. 69 days after transplantation, tumors were harvested, weighed and fixed in 10% neutral buffered formalin for 16 hours before Hematoxylin and Eosin staining. Mice were maintained under the strict guidelines of the Institutional Animal Care and Use Committee (IACUC)-approved protocols of the National Cancer Institute and National Heart, Lung, and Blood Institute.

### Cumulative cell numbers

To calculate the cumulative cell numbers, cells were split at a constant ratio (splitting ratio) for each passage. At each splitting, the cell number was counted and multiplied by the splitting ratio. The final number is the cumulative cell number. The final curve was generated by graphing the cumulative cell numbers over several passages. Note that for each cell line, a no-virus transduction control was included to determine the 0-day of counting, which is the first day when the no-virus control was completely dead after drug selection.

### ChIP-seq, ChIP, and data analyses

ChIP-seq was performed in the next generation sequencing facility at the National Cancer Institute (NCI), as previously described [18,22]. Peaks were identified by the MACS algorithm [40]. For calculating peak intensity, the number of tags at each nucleotide within a peak was calculated and summed up for all the nucleotides across the peak. The sum was defined as peak intensity, which was used to calculate the correlation between CBFB and RUNX2 binding.

To assign the peak to specific genes, we used the approach we developed previously [22]. Briefly, we arbitrarily defined the promoter region as the region between 5 kb upstream to 5 kb downstream of the transcription start site (TSS) of a transcript. The rest of the region within the gene body (5 kb downstream of TSS to the end of transcription) is defined as the gene body region. We also define regions 25 kb away from the transcript as distal.

ChIP assay were done in the same way as ChIP-seq. The amplicon enrichment was measured by realtime PCR and calculated as percentage of input. Primers for amplifying the location on the MYC gene are: forward, 5’-ACTCACAGGACAAGGATGCG-3’; Reverse, 5’-TGCTCCTCCGTAGCAGTACT-3’.

### RNA-seq and data analyses

SAOS2 cells were transduced with lentiviruses expressing shLuc, shRUNX2_3 or shRUNX2_4 for 4 days when we observed significant RUNX2 protein reduction but no obvious apoptosis. RNA-seq was performed as previously described [18]. We reasoned that at this time point, the underlying transcriptional changes precede the cellular event—apoptosis. After RNA extraction with Trizol, 1ug total RNA was subjected to deep sequencing in the NextSeq 500 machine at the NCI next generation sequencing facility. Reads were aligned to the human genome (Build hg19). The cufflinks algorithm was used to calculate reads per kilobase per million (RPKM) for each RefSeq transcript.

### Lentivirus production and transduction

shRNAs were cloned into a pLKO.1 backbone, which carries a puromycin resistant gene. For the rescue experiment, SAOS2 cells were transduced with a plenti6-GW-MYC vector and selected in 10 ug/ml of Blasticidin. Stable cells were then transduced with a RUNX2 or CBFB shRNA vector. shRNA sequences are as follows:

RUNX2(h)_3, top: 5’-CCGGTGCACTATCCAGCCACCTTTACTCGAGTAAAGGTGGCTGGATAGTGCATTTTT-3’

RUNX2(h)_3, bottom: 5’-AATTAAAAATGCACTATCCAGCCACCTTTACTCGAGTAAAGGTGGCTGGATAGTGCA-3’

RUNX2(h)_4, top: 5’-CCGGGCTACCTATCACAGAGCAATTCTCGAGAATTGCTCTGTGATAGGTAGCTTTTT-3’

RUNX2(h)_4, bottom: 5’-AATTAAAAAGCTACCTATCACAGAGCAATTCTCGAGAATTGCTCTGTGATAGGTAGC-3’

RUNX2(m)_4, top: 5’-CCGGGCAGAATGGATGAGTCTGTTTCTCGAGAAACAGACTCATCCATTCTGCTTTTT-3’

RUNX2(m)_4, bottom: 5’-AATTAAAAAGCAGAATGGATGAGTCTGTTTCTCGAGAAACAGACTCATCCATTCTGC-3’

RUNX2(m)_5, top: 5’-CCGGCCGAGTCATTTAAGGCTGCAACTCGAGTTGCAGCCTTAAATGACTCGGTTTTT-3’

RUNX2(m)_5, bottom: 5’-AATTAAAAACCGAGTCATTTAAGGCTGCAACTCGAGTTGCAGCCTTAAATGACTCGG-3’

CBFB(h)_1, top: 5’- CCGGGAGAAGCAGGCAAGGTATATTCTCGAGAATATACCTTGCCTGCTTCTCTTTTT -3’

CBFB(h)_1, bottom: 5’-AATTAAAAAGAGAAGCAGGCAAGGTATATTCTCGAGAATATACCTTGCCTGCTTCTC-3’

CBFB(h)_2, top: 5’-CCGGCCGCGAGTGTGAGATTAAGTACTCGAGTACTTAATCTCACACTCGCGGTTTTT -3’

CBFB(h)_2, bottom: 5’-AATTAAAAACCGCGAGTGTGAGATTAAGTACTCGAGTACTTAATCTCACACTCGCGG-3’

MLL1(h)_1, top: 5’-CCGGGATTCGAACACCCAGTTATTCCTCGAGGAATAACTGGGTGTTCGAATCTTTTT -3’

MLL1(h)_1, bottom: 5’-AATTAAAAAGATTCGAACACCCAGTTATTCCTCGAGGAATAACTGGGTGTTCGAATC-3’

MLL1(h)_5, top: 5’-CCGGTGCCTGGAAGGAGCCTATTATCTCGAGATAATAGGCTCCTTCCAGGCATTTTT-3’

MLL1(h)_5, bottom: 5’-AATTAAAAATGCCTGGAAGGAGCCTATTATCTCGAGATAATAGGCTCCTTCCAGGCA-3’

Menin(h)_2, top: 5’-CCGGCTGTACCTGAAAGGATCATACCTCGAGGTATGATCCTTTCAGGTACAGTTTTT-3’

Menin(h)_2, bottom: 5’-AATTAAAAACTGTACCTGAAAGGATCATACCTCGAGGTATGATCCTTTCAGGTACAG -3’

Menin(h)_5, top: 5’- CCGGTCTACGACGGCATCTGCAAATCTCGAGATTTGCAGATGCCGTCGTAGATTTTT -3’

Menin(h)_5, bottom: 5’-AATTAAAAATCTACGACGGCATCTGCAAATCTCGAGATTTGCAGATGCCGTCGTAGA-3’

MYC(h)_4, top: 5’-CCGGCCTGAGACAGATCAGCAACAACTCGAGTTGTTGCTGATCTGTCTCAGGTTTTT-3’

MYC(h)_4, bottom: 5’-AATTAAAAACCTGAGACAGATCAGCAACAACTCGAGTTGTTGCTGATCTGTCTCAGG-3’

MYC(h)_5, top: 5’-CCGGCCTGAGACAGATCAGCAACAACTCGAGTTGTTGCTGATCTGTCTCAGGTTTTT-3’

MYC(h)_5, bottom: 5’-AATTAAAAACCTGAGACAGATCAGCAACAACTCGAGTTGTTGCTGATCTGTCTCAGG-3’

### CRISPR construct and clone selection

CRISPR targeting sequences (shown in Figure) were cloned into the pX330 vector (Addgene: #42230). To delete a region in the Exon 4 of the human p53 gene, a pair of CRISPR constructs was co-transfected into the cells together with an EGFP expression vector. EGFP-positive cells were selected by flow cytometry and plated at a single cell density. Colonies were picked, propagated, and genotyped by PCR.

### Antibodies

We used the following antibodies for immunoblotting: RUNX2 (Cell Signaling, Cat:8486), p53 (DO1, Santa Cruz, Cat:sc-126), β-actin (Sigma, Cat:A5316), Cleaved PARP (Promega, Cat: G7341), Tubulin (Sigma, Cat: T3526), CBFB (Cell Signaling, Cat: 12902s), and MYC (Epitomics, Cat: 1472–1).

We used the following antibodies for ChIP: RUNX2 (house made using recombinant RUNX2 fragments), Menin (Bethyl, Cat: A300-105A), CBFB (Bethyl, Cat: A303-549A), RNA polymerase II, N20 (Santa Cruz, Cat: sc-899), H3K4me3 (Abcam, Cat: ab8580-100), H3K79me2 (Abcam, Cat: ab3594-100).

MYC immunohistochemistry was performed with a mouse monoclonal antibody of MYC, 9E11 (Santa Cruz, sc-47694).

### Menin inhibitors

Mi-2 and Mi-3 were purchased from Selleckchem, and Mi-nc from Cayman Chemical. All the inhibitors were dissolved in DMSO to make 50 mM stock solution. Working concentrations were 50 uM.

### FLAG pull-down and silver staining

We established a stable cell line for SAOS2 and Hu09-M112 cells by transducing them with the plenti6-GW-FLAG-RUNX2 vector and selecting transduced cells with 10 ug/ml of Blasticidin. After the stable cell line was established, cells from ten to twenty 10cm plates (around 60 million cells) were used to perform a FLAG pulldown. Briefly, cell pellets were lysed in 10ml of NET buffer (50 mM Tris, pH 7.5, 250 mM NaCl, 5 mM EDTA, 0.1% NP40, 10% Glycerol with freshly added protease inhibitors). Clear cell lysate was incubated with 100ul of anti-FLAG M2 affinity gel (200ul slurry) (A2220, Sigma) overnight at 4°C. FLAG_RUNX2(h) bound anti-FLAG M2 affinity gel was washed three times with NET buffer (50mM Tris, pH 7.5, 250mM NaCl, 5mM EDTA, 1% NP40, 10% Glycerol, freshly added proteinase inhibitors) and eluted four times with 500ug/ml 3xFLAG peptide (F4799, Sigma). Combined eluted materials were concentrated with acetone and resolved by sodium dodecyl sulfate-polyacrylamide gel electrophoresis (SDS-PAGE) and silver staining (SilverQuest Staining Kit, LC6070, Invitrogen). Silver stained bands were cut out from the SDS-PAGE gel and analyzed by Mass Spectrometry.

### Immunohistochemistry (IHC)

Formalin fixed paraffin embedded slides were deparaffinized in Xylene, 100% ethanol and 95% ethanol. Antigens were retrieved by boiling slides in 10 mM sodium citrate for 10 minutes. After cooling, slides were treated with 3% H2O2 for 10 minutes followed by PBS+0.1% Tween 20 washing and blocked with serum. Slides were then incubated with 1:200 MYC antibody (Epitomics) for 1 hour at room temperature, washed three times with PBS, incubated with biotinylated goat anti-rabbit IgG secondary antibody (VECTASTAIN ABC Kit) for 1 hour at room temperature. After washing with biotin-avidin solution for 30 minutes at room temperature, slides were rinsed with PBS three times, DAB solution was added to allow color development for 2–5 minutes.

### Tumor microarray (TMA) of OS

Eighty eight OS patient tumors were collected under an Institutional Review Board (IRB) approved protocol of the Montefiore Medical Center. A TMA was generated using these 88 tumors. Immunohistochemistry (IHC) procedures of using this TMA were approved under an exemption of IRB (#12806) by the Office of Human Subjects of Research (OHSR) at the National Institutes of Health.

### Masson’s trichrome staining

Formalin fixed paraffin embedded slides were deparaffinized in Xylene, 100% ethanol and 95% ethanol. Masson’s trichrome staining was performed according to the instructions of the NovaUltra Masson Trichrome Stain Kit (IHCWorld, IW-3006). Blue color indicates collagen, black color the nuclei, and red color the cytoplasm.

## Supporting Information

S1 Fig RUNX2 knockdown led to apoptosis of OS cells. (**A**) I.B. of RUNX2, cleaved PARP and Tubulin in Hu09-M112 cells. (**B**) Propidium iodide staining showing sub-G1 (apoptosis), G1, S, and G2 phases of Hu09-M112 cells. (**C**) Images of Hu09-M112 cells 6 days after virus transduction. (**D**) Cumulative cell numbers of Hu09_M112 cells transduced with lentiviruses expressing shLuc, shRUNX2_3, shRUNX2_4. 0 day is defined as the time point of 1^st^ splitting, 2 days after virus transduction. See Methods for details. (**E**) Histogram of U2OS cells transduced with lentivirus expressing shLuc, shRUNX2_3 and shRUNX2_4. (**F**) Histogram of HOS-MNNG cells transduced with lentivirus expressing shLuc, shRUNX2_3 and shRUNX2_4. (**G**) Histogram of hMSC cells transduced with lentivirus expressing shLuc, shRUNX2_3 and shRUNX2_4.
(TIF)

S2 Fig CBFB binds to RUNX2 in Hu09-M112 cells. (**A**) Flag pull-down in Hu09-M112 cells followed by silver staining. **(B**) I.B. to confirm the interaction between CBFB and RUNX2 in Hu09-M112 cells. (**C**) CBFB knockdown in hMSCs followed by I.B. of CBFB and cleaved caspase 3. (**D**) Cumulative cell number of hMSCs transduced with lentiviruses expressing shLuc, shCBFB_1 and shCBFB_2.
(TIF)

S3 Fig RUNX2 regulates the expression of MYC in OS cells. (**A**) Realtime PCR to measure the RNA levels of MYC upon RUNX2 knockdown in SAOS2 cells. (**B**) I.B. of MYC upon RUNX2 knockdown in Hu09-M112 cells. (**C**) Realtime PCR to measure the RNA levels of MYC upon CBFB knockdown in SAOS2 cells. (**D**) I.B. of MYC upon CBFB knockdown in Hu09-M112 cells. **, p<0.01; *, p<0.05.
(TIF)

S4 Fig MYC is over-expressed in and required for the survival of OS cells. (**A**) Cumulative cell number of RUNX2 knockdown rescued by exogenous MYC expression in SAOS2 cells. **(B**) Cumulative cell number of CBFB knockdown rescued by exogenous MYC expression in SAOS2 cells.
(TIF)

S5 Fig Exogenous MYC expression partially rescue the apoptosis caused by RUNX2 and CBFB knockdown. (**A**) I.B. of Myc and b-actin in mMSCs and mouse OS cell lines. (**B**) MYC immunohistochemistry of osteosarcoma TMA. Two representative tumors are shown in Fig 7D. (**C**) I.B. of MYC in Hu09-M112 cells with MYC knockdown. (**D**) Cumulative cell number of Hu09-M112 cells with MYC knockdown.
(TIF)

S1 Table RUNX2 direct targets. (XLS)

S2 Table RUNX2 bound genes. (XLS)
