# Supplementary figures and images for: A RUNX2-Mediated Epigenetic Regulation of the Survival of p53 Defective Cancer Cells
Source: PLoS Genet. 2016 Feb 29;12(2):e1005884. doi: 10.1371/journal.pgen.1005884 (PMC4771715; doi:10.1371/journal.pgen.1005884)

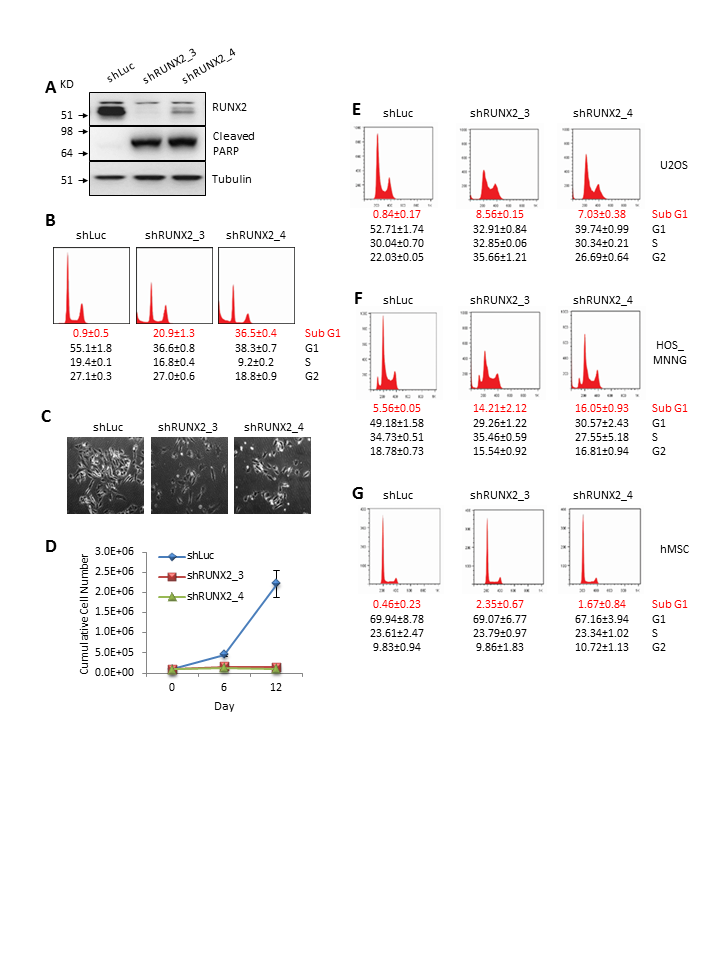

Supplement: S1 Fig — (A) I.B. of RUNX2, cleaved PARP and Tubulin in Hu09-M112 cells. (B) Propidium iodide staining showing sub-G1 (apoptosis), G1, S, and G2 phases of Hu09-M112 cells. (C) Images of Hu09-M112 cells 6 days after virus transduction. (D) Cumulative cell numbers of Hu09_M112 cells transduced with lentiviruses expressing shLuc, shRUNX2_3, shRUNX2_4. 0 day is defined as the time point of 1st splitting, 2 days after virus transduction. See Methods for details. (E) Histogram of U2OS cells transduced with lentivirus expressing shLuc, shRUNX2_3 and shRUNX2_4. (F) Histogram of HOS-MNNG cells transduced with lentivirus expressing shLuc, shRUNX2_3 and shRUNX2_4. (G) Histogram of hMSC cells transduced with lentivirus expressing shLuc, shRUNX2_3 and shRUNX2_4. (TIF) [file pgen.1005884.s001.tif]

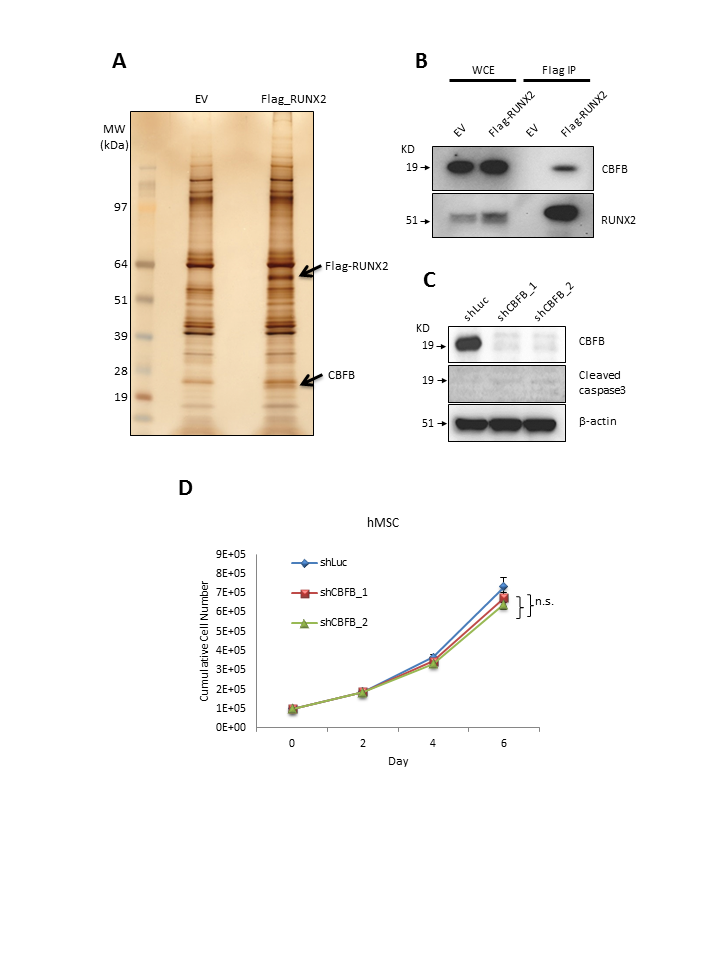

Supplement: S2 Fig — (A) Flag pull-down in Hu09-M112 cells followed by silver staining. (B) I.B. to confirm the interaction between CBFB and RUNX2 in Hu09-M112 cells. (C) CBFB knockdown in hMSCs followed by I.B. of CBFB and cleaved caspase 3. (D) Cumulative cell number of hMSCs transduced with lentiviruses expressing shLuc, shCBFB_1 and shCBFB_2. (TIF) [file pgen.1005884.s002.tif]

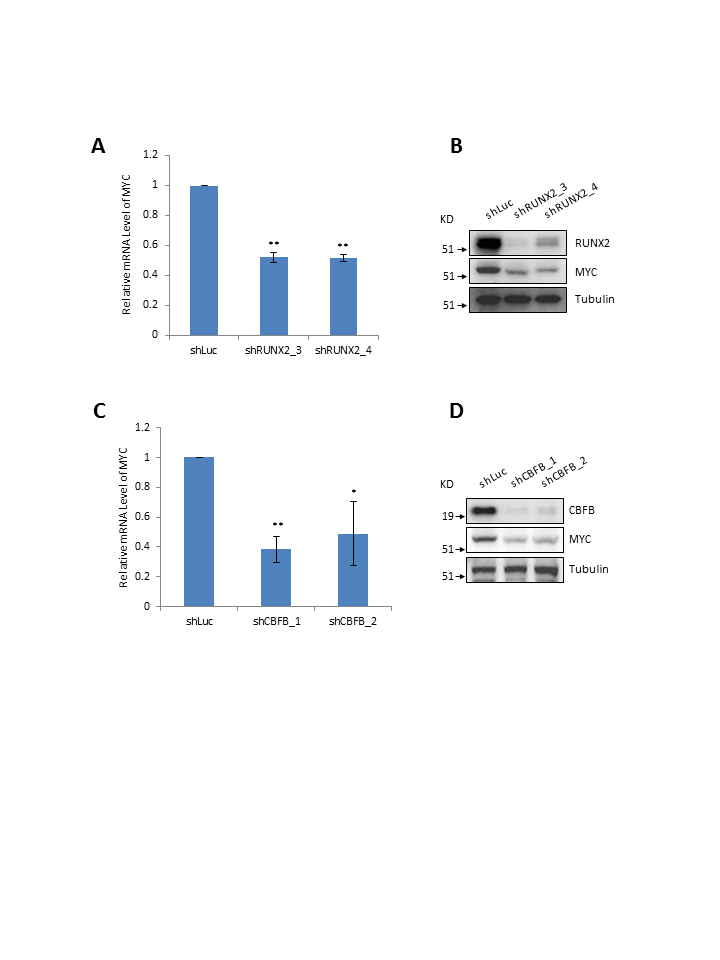

Supplement: S3 Fig — (A) Realtime PCR to measure the RNA levels of MYC upon RUNX2 knockdown in SAOS2 cells. (B) I.B. of MYC upon RUNX2 knockdown in Hu09-M112 cells. (C) Realtime PCR to measure the RNA levels of MYC upon CBFB knockdown in SAOS2 cells. (D) I.B. of MYC upon CBFB knockdown in Hu09-M112 cells. **, p<0.01; *, p<0.05. (TIF) [file pgen.1005884.s003.tif]

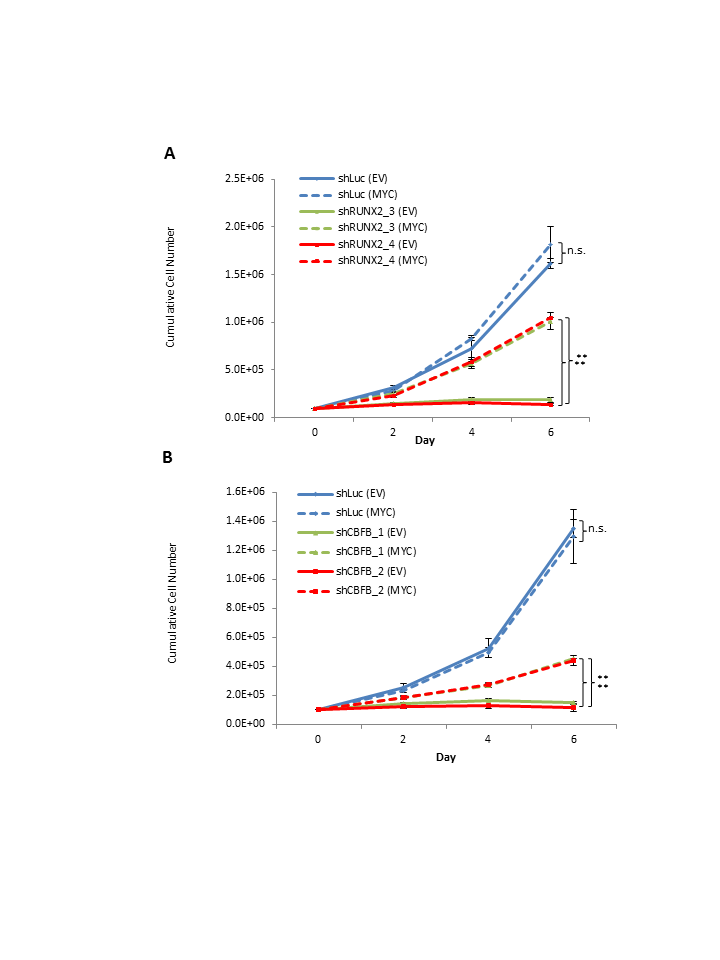

Supplement: S4 Fig — (A) Cumulative cell number of RUNX2 knockdown rescued by exogenous MYC expression in SAOS2 cells. (B) Cumulative cell number of CBFB knockdown rescued by exogenous MYC expression in SAOS2 cells. (TIF) [file pgen.1005884.s004.tif]

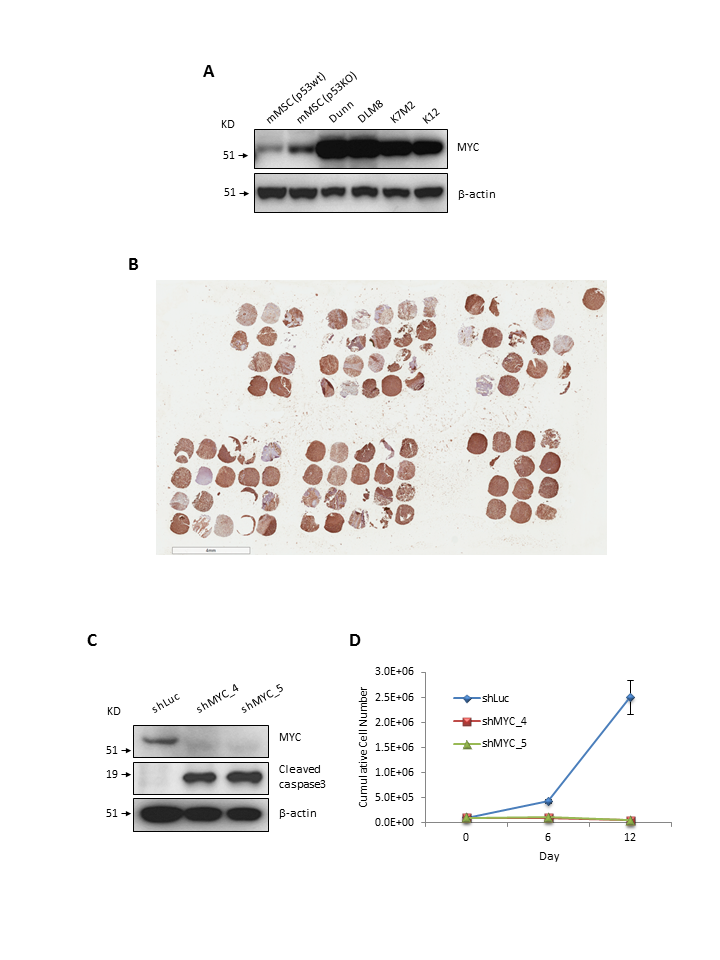

Supplement: S5 Fig — (A) I.B. of Myc and b-actin in mMSCs and mouse OS cell lines. (B) MYC immunohistochemistry of osteosarcoma TMA. Two representative tumors are shown in Fig 7D. (C) I.B. of MYC in Hu09-M112 cells with MYC knockdown. (D) Cumulative cell number of Hu09-M112 cells with MYC knockdown. (TIF) [file pgen.1005884.s005.tif]
